# Osseointegration and biocompatibility of different metal implants - a comparative experimental investigation in sheep

**DOI:** 10.1186/1471-2474-13-32

**Published:** 2012-03-08

**Authors:** Michael Plecko, Christine Sievert, Daniel Andermatt, Robert Frigg, Peter Kronen, Karina Klein, Stefan Stübinger, Katja Nuss, Alexander Bürki, Stephen Ferguson, Ulrich Stoeckle, Brigitte von Rechenberg

**Affiliations:** 1 Trauma Hospital, Goestingerstr.24, Graz, A - 8021, Austria; 2 Musculoskeletal Research Unit (MSRU), Equine Hospital, Vetsuisse Faculty, University of Zurich, Winterthurerstr.260, Zurich, CH-8057, Switzerland; 3 Synthes GmbH, Luzernerstr.19, Solothurn, CH-4500, Switzerland; 4 Competence Center for Applied Biotechnology and Molecular Medicine (CABMM), Equine Hospital, Vetsuisse Faculty, University of Zurich, Winterthurerstr.260, Zurich, CH-8057, Switzerland; 5 Institute for Surgical Technology and Biomechanics, University of Berne, Stauffacherstr.78, Berne, CH-3014, Switzerland; 6 Institute for Biomechanics, ETH Zurich, Wolfgang-Pauli-Str.10, Zurich, CH-8093, Switzerland; 7 Berufsgenossenschaftliche Unfallklinik Tübingen, Schnarrenbergstr.95, Tübingen, D-72076, Germany; 8 University Hospital Zurich, Devision of Trauma Surgery, Rämistr.100, Zurich,CH-8091, Switzerland

## Abstract

**Background:**

In the present study, 4 different metallic implant materials, either partly coated or polished, were tested for their osseointegration and biocompatibility in a pelvic implantation model in sheep.

**Methods:**

Materials to be evaluated were: Cobalt-Chrome (CC), Cobalt-Chrome/Titanium coating (CCTC), Cobalt-Chrome/Zirconium/Titanium coating (CCZTC), Pure Titanium Standard (PTST), Steel, TAN Standard (TANST) and TAN new finish (TANNEW). Surgery was performed on 7 sheep, with 18 implants per sheep, for a total of 63 implants. After 8 weeks, the specimens were harvested and evaluated macroscopically, radiologically, biomechanically (removal torque), histomorphometrically and histologically.

**Results:**

Cobalt-Chrome screws showed significantly (p = 0.031) lower removal torque values than pure titanium screws and also a tendency towards lower values compared to the other materials, except for steel. Steel screws showed no significant differences, in comparison to cobalt-chrome and TANST, however also a trend towards lower torque values than the remaining materials. The results of the fluorescence sections agreed with those of the biomechanical test. Histomorphometrically, there were no significant differences of bone area between the groups. The BIC (bone-to-implant-contact), used for the assessment of the osseointegration, was significantly lower for cobalt-chrome, compared to steel (p = 0.001). Steel again showed a lower ratio (p = 0.0001) compared to the other materials.

**Conclusion:**

This study demonstrated that cobalt-chrome and steel show less osseointegration than the other metals and metal-alloys. However, osseointegration of cobalt-chrome was improved by zirconium and/or titanium based coatings (CCTC, TANST, TAN, TANNEW) being similar as pure titanium in their osseointegrative behavior.

## Background

Osseointegration describes the direct anchorage and integration of an implant within living bone [1]. Various factors determine the progress towards osseointegration, including the implant's material properties, form and surface characteristics, mechanical load, surgical technique, location and local quality of the host bone [2]. The final goal is to reach an interface matrix, equivalent to bone in its structure, composition and biomechanical properties, to withstand early mechanical loading [3].

The mechanisms for osseointegration of metal implants are as follows: after the initial surgical lesion, through preparation of the implant bed the necrotic tissue is resorbed and new matrix is synthesized to close the gap between the bone and implant [4]. For good anchorage of the implant, primary bone healing is desired, which is characterized through direct deposition of new bone at the interface [5]. For this, immediate implant stability [6] and a minimal distance (< 1 mm) between implant and bone is a prerequisite [7]. In the early phase, a blood coagula fills the space between the implant and bone, which recruits cells for debridement and attracts multipotent mesenchymal cells from the vessels and environment [8]. These cells migrate through the coagula to the implant surface (osteoconduction) and deposit a thin, afibrillar layer on the implant [3,9]. After differentiation to osteoblasts, they deposit a collagen matrix on top of this layer and, after another 4-6 weeks, these structures are replaced by woven bone that forms the connection between the implant and surrounding bone. Over time, the woven bone is remodeled and replaced by lamellar bone with the adjacent implant firmly seated in the bone socket [7,10]. Since the implant surface had no contact to bone before, this process is also called "de novo bone formation"[11].

In order to achieve optimal osseointegration, the material properties of metallic implants are of paramount importance [12,13]. This is true for temporary and permanent implants, where both biocompatibility and mechanical endurance are of uttermost importance. For these implants, resistance to corrosion and tribocorrosion plays a role, especially if metal implants are combined, such as in plates and screws for internal fixation of bone fractures [13-15].

Stainless steel (316 L) has proven to be a good material for metal implants and was often used in trauma surgery [16]. These implants are characterized by good mechanical properties (stiffness, ductility, elasticity), easy production and low costs. However, resistance to corrosion is lower in stainless steel than in other implants such as titanium [12]. Also, biocompatibility is not optimal, primarily due to its content of nickel and the potential for an allergic reaction [17,18]. Biocompatibility is fulfilled when functionality of the implant is achieved without eliciting a foreign body reaction within the tissue [14,19]. Due to these features, stainless steel is nowadays used for temporary implants only [13]. For permanent implants cobalt chrome has replaced stainless steel mainly due to its mechanical and galvanic properties. It is mostly used in combination with molybdane (CoCrMo) [17,20]. The stiffness of CoCrMo-implants is higher than that of stainless steel and titanium and new production methods result in very fine-grained alloy compositions [15]. Corrosion resistance and fatigue of this material are very high [15,21]. However, biocompatibility and osseointegration of cobalt chrome implants are currently still under discussion [22] while titanium or its alloys shows excellent biocompatibility, osseointegration [6] with no allergenic [13] and good elastic properties [16,23,24]. Nevertheless, resistance to tribocorrosion is relatively low in titanium, due to its relatively low stiffness. Therefore, different alloys are on the market, which improve the properties of pure titanium [25]. Among them is TAN (Ti6AL7Nb), an alloy with equal corrosion resistance to pure titanium and which increasingly replaces TAV alloy (Ti6Al4V), because of the potential cytotoxicity of vanadium [25-28]. Last, like titanium the material zirconium belongs also to the heavy and reactive metals [29]. Oxide ceramics are harder, more resistant to tribocorrosion, but also more brittle in comparison to hard metals [30]. Zirconium is biocompatible, has a high ultimate breaking strength and its osseointegration is equal to titanium [31].

In this study metallic screws out of cobalt chrome coated with either titanium, TAN with different surfaces or a combination of titanium and zirconium were tested in a pelvic implantation model in sheep [32,33]. The goal was to compare these different metals for biocompatibility and osseointegration for later use in newly developed dynamic locking screws (DLS)(09.213.0.xx, Synthes^®^, Solothurn, Switzerland) [34]. The study was based on the hypothesis that cobalt chrome screws coated with titanium, TAN and/or zirconium show similar biocompatibility and osseointegration as pure titanium.

## Methods

### Implants

**Figure 1:**
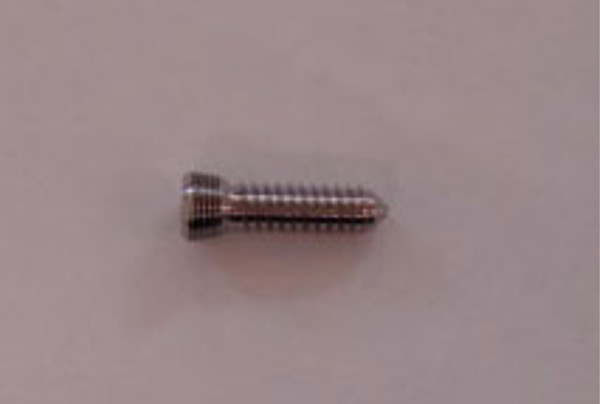
**The type of screw that was used for the all materials tested is pictured**. It was a standard, not self-tapping cortical screw design with a head fit for a SD15 Stardrive screwdriver. Screws were 14 mm in length and had a diameter of 3.5 mm with a core of 2.5 mm.

Seven different implants were used (Table 1). The design approximated a normal standard 3.5 locking screw, but without cutting flutes. Screws were 14 mm in length, with a thread diameter of 3.5 mm and a core diameter of 2.8 mm (Figure 1). Stainless steel, pure titanium and TAN (titanium alloy) were used as controls, the other materials were the groups to be tested. All threads were machine-rolled. Then surfaces were then treated as follows. For steel, the surface was electropolished leaving a surface roughness of ca. 0.22 μm. Cobalt chrome implants were treated by passivation (surface roughness ca. 0.22 μm along threads). The TAN surface was prepared by electrochemical anodisation (surface roughness of ca.0.32 μm) and the TAN-new-finish was additionally polished (surface roughness of ca. 0.28 μm). The roughness of the samples was investigated with a Nanofocus μSurf confocal microscope. The L50x objective was used and a 1 × 4 stitching applied in order to have a longer distance of measurement. All measurements were performed on the groove of the threads in the middle of the screw.

**Table 1:** The table describes the different materials tested in this study.

Material of Implant	abbreviation
Stainless Steel	Steel

Cobalt-Chrome	CC

Cobalt-Chrome/Titanium coating	CCTC

Cobalt-Chrome/Zirconium/Titanium coating	CCZTC

Pure Titanium Standard	PTST

Titanium-Aluminium-Niobium (TAN) Standard	TANST

Titanium-Aluminium-Niobium (TAN) new finish	TANNEW

Stainless steel and pure titanium were used as negative and positive controls

### Animals

Seven adult, female Swiss Alpine Sheep were used for this study. The average age was 3.3 years (2.5-4.2 years). The average weight of the sheep was 76.9 kg (51-98 kg). Animals were distributed in 3 groups with 2 animals each (n = 6) and one animal was used alone for one group. All animal experiments were conducted according to the Swiss laws of animal protection and welfare and were authorized by the local federal authorities (authorization #19/2009).

### Animal model

As reported before, the pelvic model in sheep was used to test the implants. For this the iliac wing of both sides of the pelvis was used to insert the screws [32,33]. Briefly, the screws are inserted into the pelvis along the linea glutea of the iliac wing. A total of 18 implants per group were inserted, of which 9 implants were subjected to biomechanical torque-removal test and the other 9 implants were prepared for histology with the implants in situ. Nine (9) implants per side of the pelvis were inserted, with spatially-matched screws for biomechanical and histological evaluation. Each screw type was introduced into the 9 positions of each pelvis.

### Surgery

Sheep were adapted to their new environment 2 weeks prior to surgeries. Sheep were sedated with xylazine (0.1 mg/kg BW Rompun^® ^2%, Bayer Health Care, Provet AG Lyssach, Switzerland) and Buprenorphine (0.01 mg/kg BW Temgesic^®^, Essex Chemie AG, Luzern, Switzerland). Anesthesia was induced with diazepam (0.1 mg/kg BW Valium^®^, Roche Pharma AG, Reinach, Switzerland), ketamine (3-5 mg/kg BW Narketan 10^®^, Vetoquinol AG, Belp-Bern, Switzerland) and Propofol (0.2 mg/kg BW 1% MCT Fresenius^®^, Fresenius Kabi AG, Stans, Switzerland).

Anesthesia was maintained with inhalation anesthesia (1-1.5% isoflurane (Forene^®^, Abbott AG, Baar; Switzerland)) under constant intravenous fluid application (Lactate Ringer 10 ml/kg BW/h) and propofol-infusion (1 mg/kg BW/h) using an injection pump and monitoring (pulse oxymetry, capnography, EKG, invasive blood pressure monitoring). Analgesia was achieved with an additional epidural anesthesia (morphine-HCL 0.1 mg/10 kg BW, Sintetica SA, Mendrisio, Switzerland) at the foramen lumbosacrale during surgery and injection of carprofen (4 mg/kg BW Rimadyl^®^, Pfizer AG, Zurich, Switzerland) for 4 days. Burpenorphine (0.01 mg/kg BW Temgesic^®^, Essex Chemie AG, Luzern, Switzerland) was given perioperatively and continued 3 times in 4 hour intervals. Tetanus serum (3000 IE Intervet^®^, Veterinaria AG, Zurich, Switzerland) and antibiosis (Penicillin Natrium Streuli^®^, Streuli Pharma AG Uznach, Switzerland 30000 IE/kg BW and Gentamycin - Vetagent^®^, Veterinaria AG, Zurich 4 mg/kg BW) were given as prophylaxis.

At surgery, sheep were placed in lateral recumbency with the pelvis slightly inclined (ca. 15%) towards the surgeon. The surgery was conducted as already described [33]. Briefly, an about 13 cm long curved skin incision was performed more or less parallel and in the middle of the iliac wing reaching to the acetabular region. The fascia was incised and a blunt approach to the pelvis bone was made between the middle gluteal muscle and the tensor fasciae latae muscle. With a scalpel the deep and middle gluteal muscle were incised and separated close to the iliac wing in the lower third of the muscle insertions at the iliac crest.

**Figure 2:**
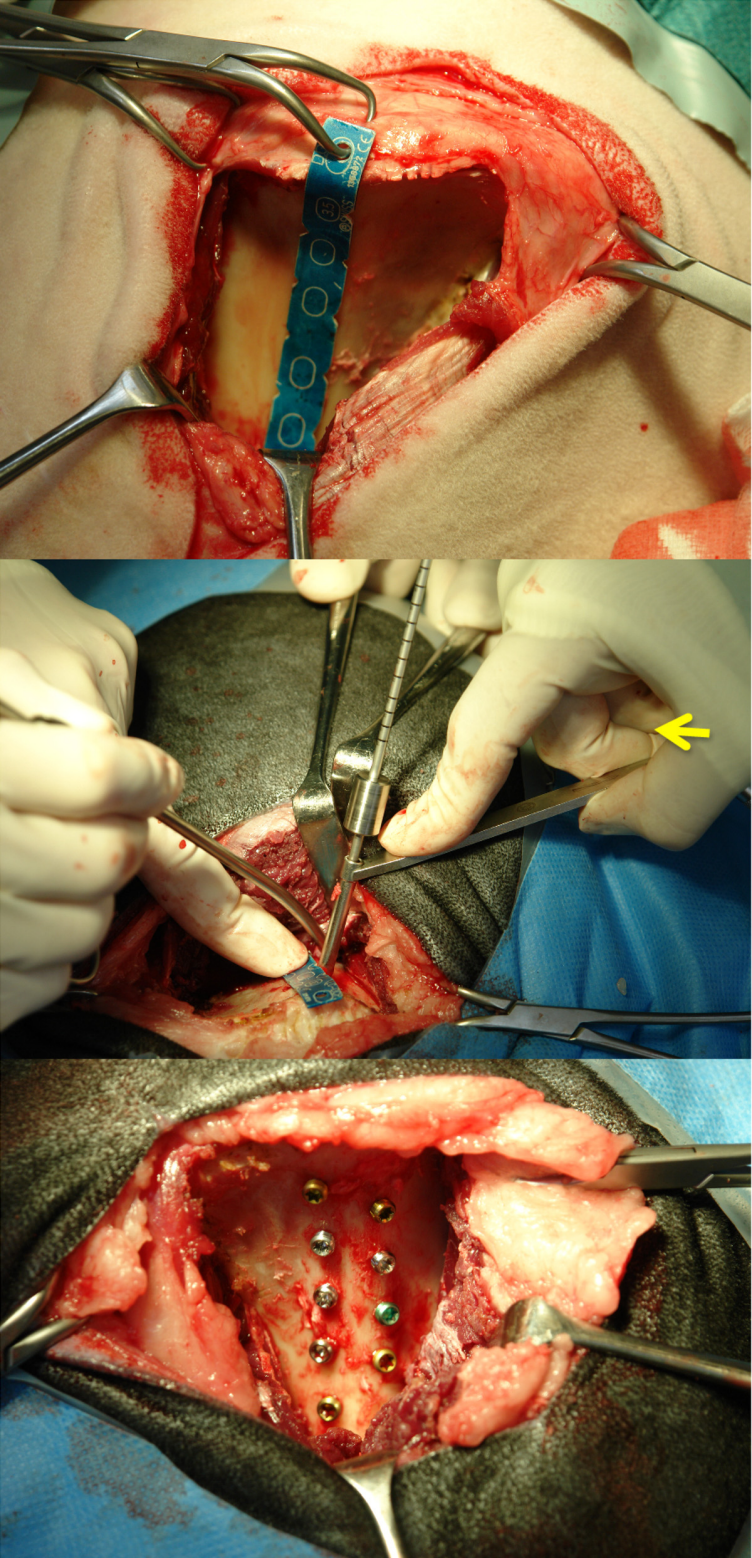
**Figure 2a shows the aluminum template in place**. The proximal end is fixed to the iliac crest. The small wedges at the sides of the template are equally spaced and alternating on the dorsal and distal site along the linea glutea of the iliac shaft. The drill hole is slightly angled (Figure 2b) to catch the most volume of cancellous bone and not to penetrate the transcortex. At the side a picture of a split pelvis demonstrates the depth of the cancellous bone along the linea glutea. All 9 screws are inserted (Fi.2c) along the linea glutea (Situs before closure of the soft tissue).

The gluteal muscles were retracted dorsally using Langenbeck and Finochietto retractors and the iliac wing was exposed. The periosteum was incised and removed ventrally, resp. dorsally exposing the entire iliac crest. A customized aluminium template with 9 drill hole markers was contoured to the linea glutea of the iliac wing with the template end joining and being fixed with a clamp right at the insertion of the gluteal muscles (Figure 2). The drill holes were prepared (2.8 mm drill bit) and tapped with a self-cutting 3.5 mm locking screw. The positions of the screws were alternating sides along the linea glutea with position 1 being most caudal and position 9 most cranial in the iliac shaft. Thereafter, the test screws were inserted and tightened with a SD15 Stardrive Screw driver. 4 screws were inserted dorsally and 5 distally to the linea glutea. Muscles were repositioned and the muscle insertions refixed with sutures to the original insertion place (Polyglactin, Vicryl^® ^2/0 Johnson & Johnson Int., Brussels, Belgium). Closure of the wound was routine. The sheep was turned to the other side and the whole surgery procedure was repeated in an identical manner on the other side.

Postoperatively the sheep were kept in small boxes for 2 weeks and then transferred to larger stalls for the remaining time of the study. After 2 months, the sheep were sacrificed.

### Intravital fluorescence markers

Fluorescence dyes were used to follow dynamic calcium deposition over time. After intravenous or subcutaneous application, these bind to calcium in the blood stream and are incorporated as hydroxyapatite crystals in newly deposited and mineralized bone matrix (12- 72 hours after application). The following dyes were used as markers: calcein green (calcein green: 10 mg/kg BW) at 4 weeks and xylenol orange (xylenol orange: 90 mg/kg BW) at 2 months, 48 hours before sacrifice. These fluorescent dyes can be detected in histology sections with a fluorescence microscope (LeicaDM6000B, Camera DFC350 FX) with the appropriate filters (L5 for calcein green, N3 for xylenol orange) giving an indication at what time new matrix was deposited over time

### Harvesting of the specimens

The pelvis was harvested immediately after slaughtering and freed from surrounding tissue. The screws were identified through scraping off partially overgrown periosteal bone from the screw heads. Tissues were assessed macroscopically for signs of inflammation and screws were checked for tight fit, respectively loosening. Findings were documented digitally as an overview of each pelvis and additionally detail pictures of each implant row were made. Radiographs (2 views, 55 kV/12 sec., 60 kV/12 sec.) of each half pelvis were made using the Faxitron (LX 60 Laboratory Radiography System^®^, faxitron x-ray corporation, Lincolnshire, Illinois, USA). With a band saw, small blocks containing the individual implants were isolated. Nine (9) corresponding implants from both sides each were either used for histology (left) or biomechanical removal torque tests (right). For the latter the specimens were wrapped in moist gauzes, sealed in plastic bags and kept cool (4°C) for 24 hours until tests were performed.

### Torque tests

To determine the removal torque, the blocks were placed in dental plaster (Dental Plaster GC Fujirock^® ^EP, GC Europe, Leuven, Belgium) in square forms. After setting of the plaster, the implant heads were connected the actuator of a servohydraulic test machine (MTS Mini Bionix 858, MTS Systems Corporation, Eden Prairie, USA). The plaster-block was lowered into an aluminum form, which was filled with an alloy (Ostalloy 117, Metallum AG Pratteln, Switzerland - melting point at 47°C). The setting of the alloy stabilized the plaster block and ensured implant alignment with the rotational actuator. Removal torque testing was performed by rotating the actuator counter-clockwise at 0.1 degrees per second. The curve for the torque vs. rotation angle was documented and evaluated. The removal torque value was defined as the maximum torque observed for curves with a clear peak. For curves without a clear peak, a straight line was constructed parallel to the linear portion of the curve, offset by 0.72 degrees (0.2% full rotation) and the intersection of this line with the torque-rotation curve was defined as the failure/yield torque. After loosening, the screws were removed and placed in 70% ethanol. The torque removal values (Nmm) were determined with a custom algorithm (Matlab, The MathWorks Inc.).

### Histology

**Figure 3:**
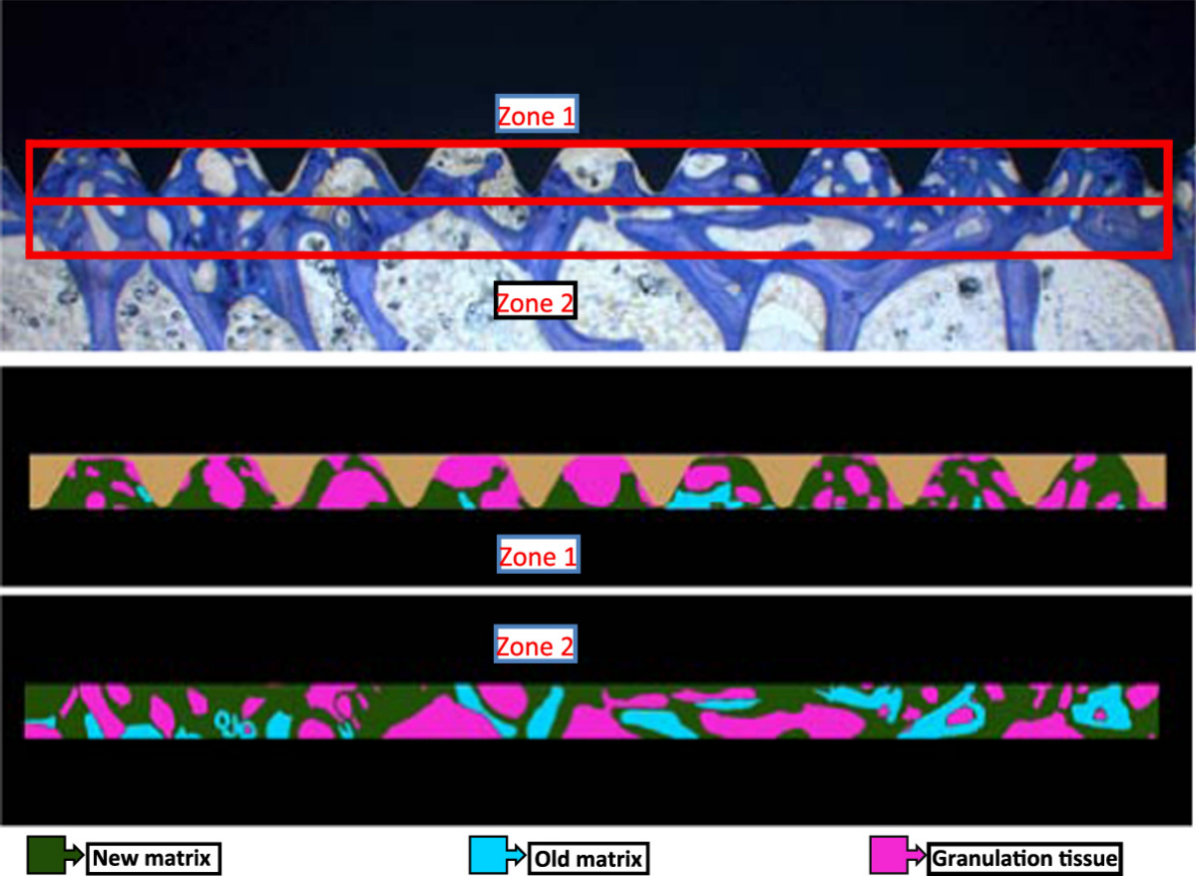
**The zone directly adjacent to the implant was measured within the thread part, whereas the zone far to the implant extended for the same size into the adjacent trabecular bone**.

**Figure 4:**
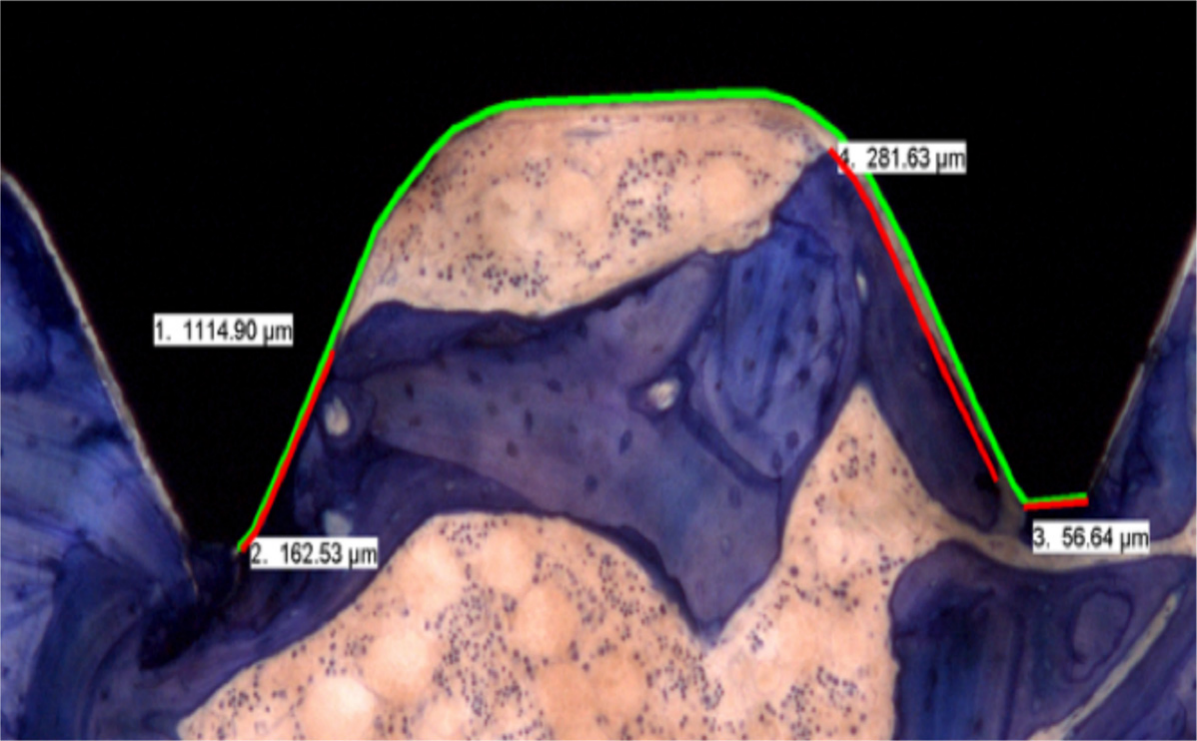
**The BIC measurements are demonstrated in this picture**. The green line indicates the total length of the thread measured. The red line measured the direct contact between bone and implant. Final results were given as percentage of BIC to the total area measured.

Specimens were fixed in 40% ethanol at 4°C and further dehydrated in an ascending series of ethanol (50%, 70%, 80%, 90% 96%, 100%) before being defatted in xylene under vacuum [35]. Probes were cut parallel to the implant axis such that serial cuts could be performed after embedding and an exact splitting of the implants along the long axis was achieved. Embedding of specimens in methylmetacrylate (Methacrylacid-methylester - Fluka Chemie GmbH, Buchs, Switzerland; Dibutylphthalat - Merck-Schuchardt OHG, Hohenbrunn, Germany; Perkadox 16 - Dr. Grogg Chemie AG, Stetten, Switzerland) was performed in customized teflon forms. After polymerization, the blocks were mounted on plastic frames and cut with a precision saw (Leica SP 1600^®^, Leica Instruments GmbH, Nussloch, Germany) or with a special band saw equipped with a diamond saw blade (EXAKT Band System 300/301^®^, Norderstedt, Germany). Ground sections were mounted on acropal slides (Perspex GS Acrylglas Opal 1013, Wachendorf AG, Basel, Switzerland) and polished to 40- 50 μm sections (Exakt^® ^Mikroschleifsystem 400 CS, Exakt Apparatebau GmbH, Norderstett, Germany), while native sections (350 μm) were glued on transparent plastic slides and wrapped in aluminum foil to preserve fluorescence. Ground sections were surface stained with toluidine blue. Ground sections were evaluated qualitatively for their osseointegration, new bone formation and bone resorption. Quantitative evaluation was performed with histomorphometry. Sections were digitally recorded with a macroscope (Leica M420, Camera DFC 320, Leica Microsystems, Heerbrugg, Switzerland; magnification 0.5 × 8) using a specialized software (Leica, IM 1000 Image manager). Both sides of the implants were digitized, visualizing the bone-implant interface at the threads. Using a specialized software program (Adobe Photoshop 3.0), zones around the implants were framed (Figure 3) such that the area of new bone or resp. granulation/fibrous tissue formation could be measured close to the implant and within threads (zone 1) as well as adjoining the implant (zone 2). The various tissues were detected manually using the Adobe Photoshop program giving each fraction a different color (new bone, old bone matrix, granulation/fibrous tissue). Old and new bone matrix could be distinguished according to color (light blue = old matrix, dark blue = new matrix). These fractions were then later measured with a special software image analysis software (QIPS/QWIN, Leica standard, V.3.0, 2003) and a standardized macro-routine using binary segmentation and the different fractions were automatically detected measured in pixels. Results were exported into a spreadsheet (Excel, Microsoft Office 2003) where the percentage of each fraction/total tissue volume and zone was calculated. For measuring bone to implant contact (BIC) the interface was digitalized at a higher magnification (0.5 × 10) and with a special measurement tool the entire surface of the implants was measured except the tip of the screws. Thereafter, the actual contact line between bone and implant was measured separately on each side (Figure 4). The difference consisted of granulation/fibrous tissue adjacent to the implant. This calculation was done for each thread separately amounting to 22 sectors per screw. When measures were completed, the BIC was calculated for the entire implant and the total value was used for statistical evaluation. In some cases, where threads were not measureable, the total surface was calculated accordingly.

*Fluorescence *was evaluated with a fluorescence microscope (Leica DM6000 B, Camera: DFC350 FX, 12.5 × magnification). Two pictures per implant were recorded including the screw head or the tip of the implant. Overlay pictures showed the different colors visualized with special filters for calcein green (L5) and xylenol orange (N3). Intensity and distribution of fluorescence were evaluated and compared as well as the direct contact and nature of fluorescent areas and implants.

### Microradiographs

Before mounting on the slides, the sections were radiographed with the Faxitron (Cabinet xray faxitron series, model 43855 A, Hewlett Packard, Mc Miniville Division Oregon USA; 28 kV, 11 sec.) on high resolution films (Fuji Photo Film Co^® ^ltd. Tokyo, Japan, Type PII for Linac/Oncology 25.7 × 30.5 cm).

Micrographs were evaluated for bone sclerosis, bone resorption including gaps between implant and bone.

*Statistical evaluation *of biomechanical tests and histomorphometrical measurements was performed using the SPSS software (SPSS Statistics, Version 17.0). Mean values and standard deviations were calculated. A factorial analysis of variance (ANOVA) was used to test for statistically significant differences, with Bonferroni post-hoc tests for inter-group comparisons. Significance was set at p < 0.05.

## Results

*Surgeries *went well for all animals and recovery was uneventful. Implants could be inserted without problems. After 2 days all sheep were ambulating without lameness and showed normal food and water intake. One screw hole in position 1 was set too far caudally and thus had to be loaded with a position screw. An additional screw hole was drilled to accommodate the implant originally planned for position 1. One screw in position 9 was tightened too much and stripped and one screw could not be fully inserted (1 TANST). These 3 screws were removed from statistical evaluation. At the end 60/63 could be included in the evaluation.

**Figure 5:**
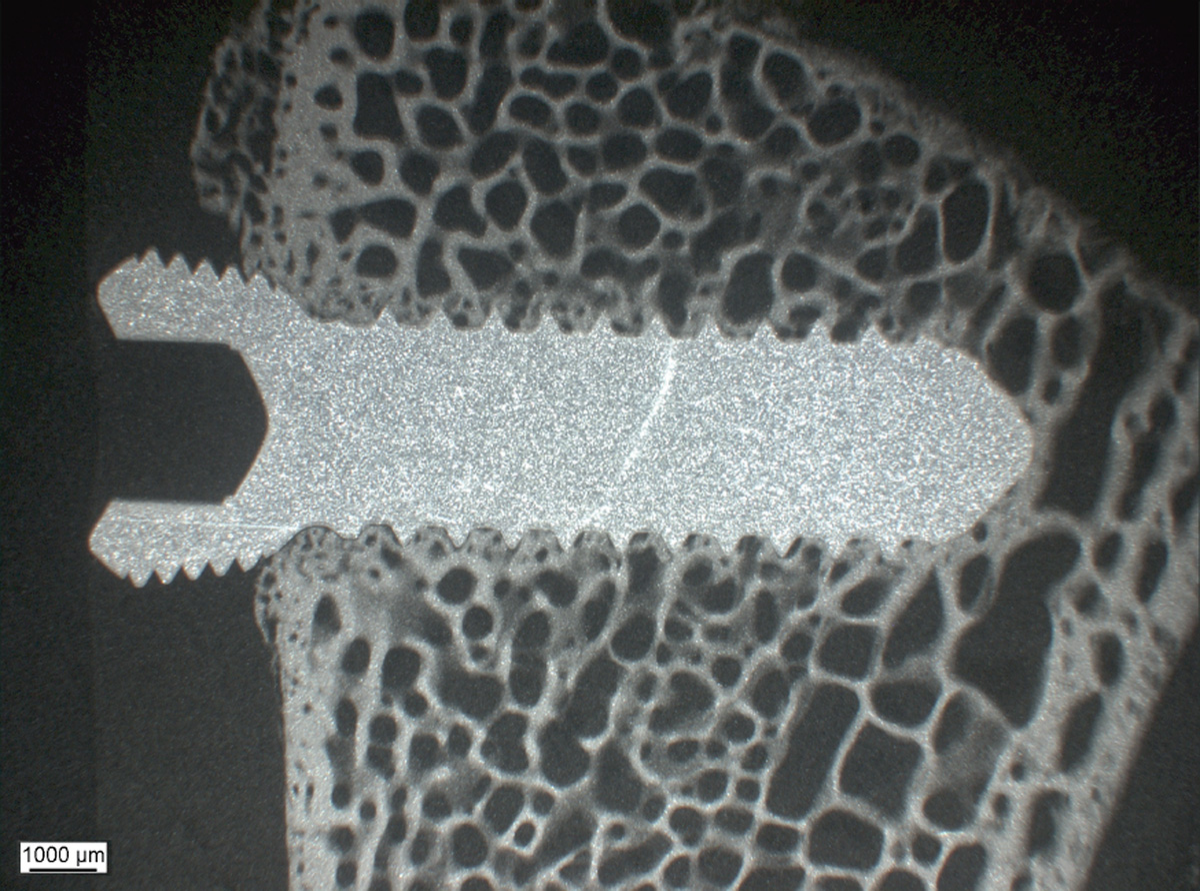
**The microradiographs allow visualization of calcified area, resp**. bone resorption along the implant. Most of the specimens showed a slight increase in bone density along the implant and no bone resorption.

*Macroscopic and radiological evaluation *revealed firm seats of all implants with implants in the proximal part of the iliac bone being mostly covered with new periosteal bone. No signs of inflammation and/or osteolysis were noticed. Radiographs showed correct seat and no osteolytic seam around the implants. Microradiographs showed no resorption zone around the implants for all groups (Figure 5).

### Removal torque tests

From the 60 screws, 3 implants had to be excluded because they were either loose (2 for CC group), or overgrown by new bone (1 for CCTC). A significantly lower removal torque value was found for chrome cobalt screws (p = 0.031) compared to pure titanium screws, but not to steel. All other 5 implants showed comparable torque values. However, there was a tendency for both the cobalt chrome and steel screws to show lower values compared to all other groups. Screws in the positions 1-5 showed slightly higher torque values compared to those of position 6-9, although no statistical differences were present. Furthermore, no significant differences were found between the right and left side of the pelvis. Values are given in Table 2.

**Table 2:** Results of the torque removal tests are shown as mean values and standard deviations in Nmm.

Type of screw	Removal Torque in Nmm	**std. dev**. ± in Nmm	N
PTST	270.9	87.3	9

TANNEW	259.9	100.1	9

CCZTC	246.6	96.9	9

CCTC	245.6	91.1	8

TANST	234.0	55.8	9

Steel	155.8	83.6	9

CC	130.1	45.8	7

Note that Steel and cobalt-chrome have the lowest values compared to all other titanium or zirconium based implants. Statistically significant differences were found only for chrome cobalt compared to pure titanium screws (p = 0.031) (N = number of screws tested per group)

### Histology

Because of the hardness of cobalt chrome screws, sections of these implants had to be cut with the EXAKT band saw instead of the precision saw. Therefore, in the absence of a precise aiming device not all screws were exactly cut in the middle of the longitudinal axis of the screws. Also, the loss of material was higher due to the thickness of the diamond band and thus, not all sections could be evaluated. In one instance, the screw loosened during the sawing process. However, the BIC could still be evaluated since the interface tissue was well preserved in the plastic sections.

**Figure 6:**
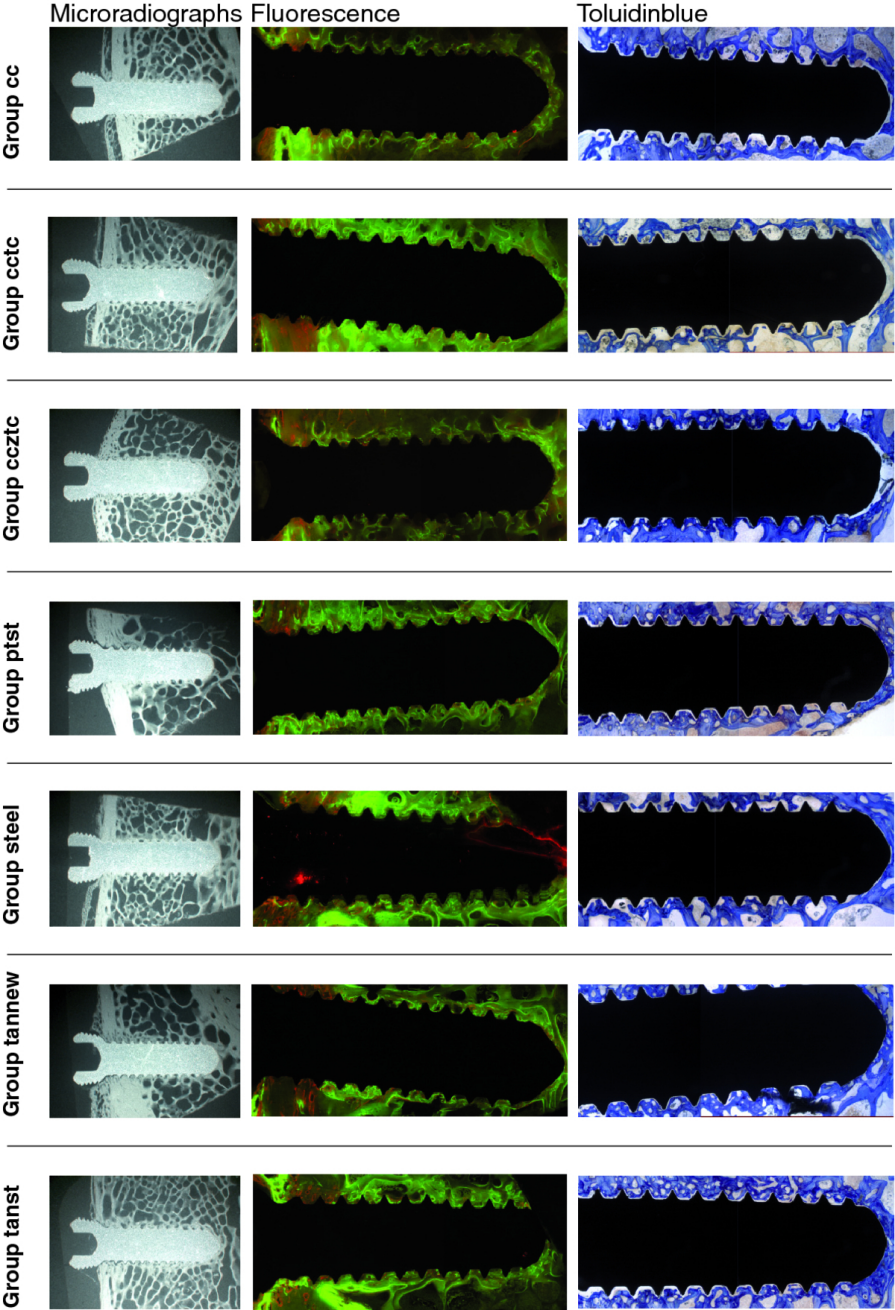
**An overview of ground sections (PMMA embedding, surface staining with toluidine blue), native fluorescence sections and microradiographs is given for all groups**. Note the similar appearance for all titanium based or zirconium or titanium coated implants. Differences exist for steel and cobalt chrome implants.

All implants showed good osseointegration (Figure 6), although for cobalt chrome and steel screws, less new bone was directly deposited on the implant. Direct contact healing was evident, with no areas of secondary bone healing. There were no qualitative differences in bone healing between groups. New bone formation, respectively remodeling at the insertion site, was similar in all groups and also between cortical and trabecular bone. Cellular reaction to implants was minimal and no mononuclear cell accumulations (lymphocytes, monocytes) or increase of multinuclear giant foreign body cells or osteoclasts were found. Therefore, statistical analysis of cellular reactions was not performed.

Histomorphometrical measurements (Table 3) showed no significant differences in old or new bone matrix between groups and within zones. The percentage of new bone and granulation tissue was higher in the zone between threads and closer to the implant compared to the adjacent bone. In contrast, the percentage of old bone matrix was higher in the bone adjacent and further away to the implant, although not with statistical significance..

**Table 3:** Histomorphometrical measurements of old and new bone matrix and granulation tissue are given in percentage of measured area.

Type of screw	Zone*	old matrix %	new matrix %	granulation tissue%
CC	Z1	4.5 ± 3.8	38.6 ± 7.1	56.9 ± 8.8

	Z2	17.9 ± 7.5	43.9 ± 8.5	38.2 ± 11.7

CCTC	Z1	7.0 ± 6.8	42.8 ± 9.9	50.2 ± 13.5

	Z2	20.9 ± 12.1	41.7 ± 6.1	37.4 ± 11.7

CCZTC	Z1	5.8 ± 4.1	44.6 ± 10.5	49.6 ± 12.3

	Z2	15.5 ± 8.0	42.7 ± 9.6	41.8 ± 11.6

PTST	Z1	5.2 ± 5.5	45.2 ± 8.8	49.5 ± 11.1

	Z2	18.2 ± 9.4	39.5 ± 10.6	42.3 ± 10.3

Steel	Z1	6.0 ± 5.5	45.5 ± 12.5	48.6 ± 15.1

	Z2	18.0 ± 9.9	42.4 ± 9.6	39.6 ± 9.6

TANNEW	Z1	6.6 ± 6.0	47.8 ± 11.5	45.6 ± 11.2

	Z2	16.3 ± 7.9	41.7 ± 10.6	42.0 ± 13.2

TANST	Z1	4.7 ± 4.5	46.2 ± 8.1	49.2 ± 9.1

	Z2	17.7 ± 7.8	41.3 ± 6.3	41.0 ± 9.7

The values are given corresponding to zones (Z1 = adjacent to implant, Z2 = far from implant). All implants show similar values with no significant differences found between groups. Values measured for granulation tissue correspond with those found in normal bone, while the relatively high percentage of new bone matrix indicates new bone formation and activity of osseointegration. The largest difference is seen for the old bone matrix indicating that bone resorption as an answer to the original surgical trauma is more intensive close to the implant. Also the granulation tissue is less in the zone farther away from the implant

*Z1 = close to implant Z2 = far from implant

The measurements of BIC (Table 4) revealed statistically significant differences between cobalt chrome and steel (p = 0.001) and all other groups (p < 0.0001).

**Table 4:** The bone-implant-contact (BIC) measurements of threads is demonstrated.

Type of screw	BIC in %	± in %
CCZTC	39.1	22.4

TANNEW	38.0	23.5

PTST	36.5	23.5

TANST	34.8	22.0

CCTC	33.6	23.1

Steel	23.2	18.8

CC	14.0	13.6

Note that steel and cobalt chrome have significantly less contact compared to all other type of materials. The chrome based implants either coated with zirconium (CCZTC) or titanium (CCTC) show equal BIC values than the other titanium based screw implants

### Fluorescence

Fluorescence markers were detected in all specimens and were distributed over the entire surface area of the implants. The width of the reaction into the adjacent bone extended about 1.5 of the length of the threads. In all implants calcein green depositions were mainly found within the trabecular and xylenol orange in the cortical area. Steel and cobalt chrome implants showed less direct marker deposition directly at the bone-implant interface compared to all other groups, which revealed a distinct, sharply demarcated well fluorescent interface.

In summary all groups showed good biocompatibility in this study. Differences were found mainly between cobalt chrome and steel implants and all other groups, but not between groups with titanium or coated with TAN, TAN-new finish or zirconium/titanium). Significant differences were found for torque values between cobalt chrome and titanium screws (p = 0.031), while only a tendency was noticed for steel screws. However, if cobalt chrome screws were coated with zirconium and/or titanium, osseointegration was considerably improved and similar to titanium screws. The new titanium alloys TAN standard and TAN new finish showed an equivalent osseointegration behavior to pure titanium. Histomorphometrical measurements of old resp. new bone matrix and granulation tissue revealed no significant difference neither for groups nor zones. BIC measurements were significantly lower in cobalt chrome screws compared to steel (p = 0.001) and all other groups (*p *< 0.0001).

## Discussion

In this study an animal model with sheep was chosen, where the pelvis served well for comparison of osseointegration between different implant groups [32,33]. As sheep have similar bone metabolism as humans, results can be accepted as indicative also for later use in humans [36-39]. As already demonstrated [33], the pelvic model allows excellent comparison for osseointegration between but also within individual animals. Insertion in the pelvis facilitates comparison between cortical and trabecular bone beds. The outcome of this study confirmed our earlier results showing that no significant differences were present between positions of screws and comparison of right and left side of the pelvis. The follow-up of 8 weeks was chosen, since early wound healing due to surgery is already completed, but foreign body reactions are still ongoing at this time period. Furthermore, osseointegration is already at a high level at this time point.

Results of removal torque tests were according to the expectations from the literature [21,33] with cobalt chrome and steel showing lower values compared to titanium or its alloys. Coating cobalt chrome screws with titanium (TAN standard and new finish) and zirconium improved removal torque to similar values than the other titanium and titanium alloy implants, indicating that these materials lend their biocompatibility and osseointegration behavior to the coating finish [40]. Pearce et al. [41] compared electropolished TAN standard in the tibia of sheep to the conventionally used TAN standard, with normal surface roughness, and to steel. The electropolished variant of TAN had a tendency for lower torque values, although similarly to the TAN standard and TAN new finish in our study, differences were not statistically significant. This was in contrast to steel that showed significantly lower values similar to another study, where in minipigs steel had significantly lower values than TAV implants [24,41]. As roughness of the metallic surface is correlated to the quality of osseointegration, the smooth and polished surfaces of cobalt chrome and steel screws correlated well to the relatively lower torque values, compared to pure titanium or zirconium or titanium coated screws. Most of the commercially available orthopedic implants have a moderate surface roughness. Nevertheless, especially those for osteosynthesis have a limited roughness, since they are normally removed after bone healing and too much osseointegration would be counterproductive for implant retrieval [6].

Histology results supported findings obtained with removal torque tests, such that BIC corresponded well: the higher BIC the higher the removal torque values and vice versa. Histomorphometrical measurements of new and old matrix, resp. granulation tissue, were similar in all groups, although more new bone was found in the zone close to the implants compared to the adjacent bone. These findings were similar to other studies [24,42] and were also nicely demonstrated also with fluorescent markers, where implants showed direct apposition of new bone close to the implant and within the adjacent bone, especially in pure titanium or surface coated implants (PTST, TAN. TANNEW, CCTC, CCZTC). For CC and steel implants the direct apposition of markers at the implant was less marked. The sequential deposition of markers showed that new bone formation at 4 weeks occurred mainly within the trabecular and at 8 weeks within the cortical bone.

*In vitro *experiments revealed that osteoblasts grew faster on titanium compared to cobalt chrome [43]. This may explain why BIC values in the current study are considerably lower in cobalt chrome than in all groups with titanium surfaces. BIC were evaluated on one section, since screws could only be cut in one plane. If another plane were cut, a more threedimensional picture of the BIC would have been obtained. This was not possible due to the size of the implants and the relatively high loss of material during sawing. However, all threads of the implant were evaluated and total BIC values were calculated. This resulted in a sound average of BIC per implant/threads and it is questionable whether values would have changed if a more 3-D approach would be possible. Since results were comparable to other studies in the literature, it is safe to assume that they were reliable and reflect clinical reality.

BIC measurements and also results of fluorescence, indicating the location and sequence of calcium deposition along the implant, supported the results of removal torque tests, as was detected also in other studies [24,44]. Primary lamellar bone was attached at the surface of the implants, where fluorescence was also visualized directly adjacent to the surface as in the current study. Titanium surfaces including TAN standard and electropolished surfaces always demonstrated higher BIC values compared to steel [24,41], although electropolished TAN had lower removal torque values [41,45] and soft tissues were easier to remove *ex vivo *from plates and locking screws [45]. In our study only screws were used and no differences were noticed between groups when screw heads had to be freed from periosteal overgrowth.

Cobalt chrome implants had significantly lower torque and BIC values. What may be a disadvantage for permanent implants like hip or knee prosthesis may be an advantage for temporary implants. With the excellent properties against fretting corrosion, cobalt chrome may be an attractive alternative for temporary implants under high mechanical load. If excellent osseointegrative properties are warranted in permanent implants, coating with TAN standard, TAN new finish or zirconium may prove to be very successful. However, long-term results are missing where the durability of such coatings under high mechanical load and its galvanic corrosion properties in direct contact with other metal implants (e.g. plate and screws) need to be tested. Additional studies with special implants for dynamic locking screws [34] are underway and will answer this question in a fracture model in sheep.

## Conclusion

Results of this study demonstrated good biocompatibility of all titanium or zirconium coated materials and combinations thereof. The surface coating of cobalt chrome implants with titanium or zirconium/titanium increased their overall osseointegration and makes them highly attractive material combinations for orthopedic implants by combining excellent mechanical and osseointegrative properties.

## Abbreviations

CC: Cobalt-chrome; CCTC: Cobalt-chrome/titanium coating; CCZTC: Cobalt- Chrome/zirconium/titanium coating; PTST: Pure titanium standard; Steel: Stainless steel; TAN alloy: Titanium-aluminum-niobium alloy (Ti6AL7Nb); TAV alloy: Titanium-aluminum-vanadium alloy (Ti6Al4V); TANST: TAN Standard; TANNEW: TAN new finish; BIC: Bone-to-implant-contact

## Competing interests

The authors have no competing interests in this study, except industrial partners (DA, RF, RK) who produced the implants and are employees of the company Synthes GmbH, Solothurn, Switzerland, which financed the entire study.

## Authors' contributions

MP: surgery, design and evaluation of the study. CS: doctorate student. DA: implant design and production. RF: implant design and production. RK: implant design and production. NF: anaesthesia and animal care. PK: anaesthesia and analgesia. KK: surgery, animal care, histology evaluation, statistical evaluation. KN: surgery, animal care, histology evaluation. AB: biomechanical testing and evaluation. SF: biomechanical testing and evaluation. US: study design, evaluation and interpretation of results. BvR: surgery, study design, evaluation and interpretation of results, leader of experimental study. All authors read and approved the final manuscript.

## Disclosures

The manuscript is an extract of the doctorate thesis of CS submitted to the University of Zurich (in German), 2011.

## Pre-publication history

The pre-publication history for this paper can be accessed here:

http://www.biomedcentral.com/1471-2474/13/32/prepub
